# Moving from a regional to a continental perspective of *Phragmites australis* invasion in North America

**DOI:** 10.1093/aobpla/pls040

**Published:** 2012-12-04

**Authors:** Karin M. Kettenring, Sylvie de Blois, Donald P. Hauber

**Affiliations:** 1 Department of Watershed Sciences and Ecology Center, Utah State University, Logan, UT 84322, USA; 2 McGill School of Environment and Department of Plant Science, McGill University, 21 111 Lakeshore, Ste-Anne-de-Bellevue, QC H9X 3V9, Canada; 3 Department of Biological Sciences, Loyola University, New Orleans, LA 70118, USA

## Abstract

**Aims:**

We use a regional comparison of *Phragmites australis* (common reed) subsp. *americanus*, *P. australis* subsp. *berlandieri* and introduced *P. australis* (possibly five sublineages) in the Chesapeake Bay, the St Lawrence River, Utah and the Gulf Coast to inform a North American perspective on *P. australis* invasion patterns, drivers, impacts and research needs.

**Findings and research needs:**

Our regional assessments reveal substantial diversity within and between the three main lineages of *P. australis* in terms of mode of reproduction and the types of environment occupied. For introduced *P. australis*, the timing of introduction also differed between the regions. Nevertheless, a common finding in these regions reinforces the notion that introduced *P. australis* is opportunistic and thrives in disturbed habitats. Thus, we expect to see substantial expansion of introduced *P. australis* with increasing anthropogenic disturbances in each of these regions. Although there have been some studies documenting the negative impacts of introduced *P. australis*, it also plays a beneficial role in some regions, and in some cases, the purported negative impacts are unproven. There is also a broader need to clarify the genetic and ecological relationships between the different introduced sublineages observed in North America, and their relative competitive ability and potential for admixture. This may be done through regional studies that use similar methodologies and share results to uncover common patterns and processes. To our knowledge, such studies have not been performed on *P. australis* in spite of the broad attention given to this species. Such research could advance theoretical knowledge on biological invasion by helping to determine the extent to which the patterns observed can be generalized or are sublineage specific or region specific.

**Synthesis:**

Given what appears to be sometimes idiosyncratic invasion patterns when interpreted in isolation in the regions that we analysed, it may be time to consider initiatives on a continental (if not intercontinental) scale to tackle unresolved issues about *P. australis*.

## Abstract (precis)

Here we describe the results of a regional comparison of introduced *Phragmites australis* and two other *P. australis* lineages found in North America. The regional similarities and differences in introduced *P. australis* invasion highlight the importance of continental-scale studies for decoding plant invasions.

## Introduction

Understanding the patterns, drivers and impacts of plant invasions requires a perspective that is both detailed and broad as well as coordinated efforts to collect, store and access data (Lodge *et al.* 2006; Mack *et al.* 2007). However, for many species we lack these different levels of research and assessment to allow for synthesis and consensus on what makes invasive species successful and on their real impacts on ecosystems. To advance our understanding of the invasion process, we suggest that an effective approach is to focus on model species that are intensively studied at multiple scales in an attempt to synthesize knowledge (e.g. *Bromus tectorum*; Novak and Mack 2001).

We can apply such an approach to the invasion of *Phragmites australis* (common reed) in North America. *Phragmites australis* is a globally distributed species consisting of a number of described subspecies, lineages and sublineages. It is emerging as a model system for studying invasive plants in North America, in part because of the phenotypic and ecological diversity within and between the lineages. For the reader to better appreciate the invasion of *P. australis*, it is best to begin with the terminology and taxonomy that is used in this review and explain how it relates to recent literature, all the while realizing that the taxonomy is in flux as more genetic and biogeographic data are accumulated.

The major phylogenetic groups of *P. australis* are referred to as ‘lineages’. In North America there are currently three major lineages that have been recognized: native *P. australis* subsp. *americanus*, *P. australis* subsp. *berlandieri* and introduced *P. australis*. The introduced lineage, first described by Saltonstall (2002), is one of the most invasive plants in North American wetlands (Marks *et al.* 1994; Galatowitsch *et al.* 1999) and has been extensively studied in many regions. In this review, secondary genetic clustering within the lineages is referred to as ‘sublineages’. ‘Haplotype’ refers to a particular set of sequences from the chloroplast (cp) DNA; individuals that share the same haplotype share the same set of cpDNA sequences. Sublineages, defined by both the haplotype and nuclear genotype, have been described for the introduced lineage only. Table 1 helps illustrate how the names and terms used in this review relate to those in the literature.

**Table 1:** Lineages, sublineages and haplotypes of *P. australis*.

Lineage^a^	Sublineages^b^	Haplotypes
Native *P. australis* subsp. *americanus*	None identified	A-H, S, Z, AA, AB, AC, E1, E2, E3, E4^c,d,e^
*P. australis* subsp. *berlandieri*^f^ (Land,^g^ Gulf Coast type^h^)	None identified	I
Introduced *P. australis*	Short B^f^ or EU^g^	M
	Short A^f^ or Greeny 1^g^	M
	Delta^f,g^	M1
	Greeny 2^g^	AD
	Greeny 3^g^	AI

^a^For a summary of possible origins and North American ranges of these lineages, see Meyerson *et al.* (2012).

^b^In two situations, multiple names were given to the same sublineage by independently operating research groups.

^c^Saltonstall (2002).

^d^Saltonstall *et al.* (2004).

^e^Meadows and Saltonstall (2007).

^f^Hauber *et al.* (2011).

^g^Lambertini *et al.* (2012).

^h^Pellegrin and Hauber (1999).

Among the introduced sublineages, much of the current understanding of the genetic relationships comes from two recent studies of introduced *P. australis* populations in the Mississippi River Balize delta on the US Gulf Coast (Hauber *et al.* 2011; Lambertini *et al.* 2012). Based mostly on microsatellite analysis in conjunction with haplotype sequencing, most introduced populations along the east coast of North America and the Great Lakes region align with the Short B sublineage (Hauber *et al.* 2011; a.k.a. EU, Lambertini *et al.* 2012). Interestingly, the Balize delta is dominated by a unique sublineage, Delta (Hauber *et al.* 2011; Lambertini *et al.* 2012), while Short B is relatively uncommon there. The other introduced sublineages listed in Table 1 have all been found in the Balize delta but are also relatively uncommon (Lambertini *et al.* 2012). Greeny 2 and 3 have not been found elsewhere in North America, and Short A (Greeny 1) is rare outside of the Balize delta (Lambertini *et al.* 2012). Further details of the Balize delta sublineages are found in the discussion on the Gulf Coast region later in this review. Whereas the haplotype diversity of the native *P. australis* subsp. *americanus* had been known for a while, it is only recently that studies have reported on the considerable genetic and phenotypic diversity in the introduced lineage.

So far, most ecological studies on *P. australis* in North America, including the ones reviewed here, have been conducted at the landscape or regional scale and have compared ecological patterns at the broad lineage level (i.e. native vs. introduced type). As knowledge continues to improve on the genetic composition of lineages and sublineages of *P. australis*, it is important that researchers compare ecological and genetic patterns across regions to highlight similarities and differences. Such a continental perspective can also help identify biases in research focus, locate gaps in knowledge and provide direction for future research initiatives.

**Fig. 1:**
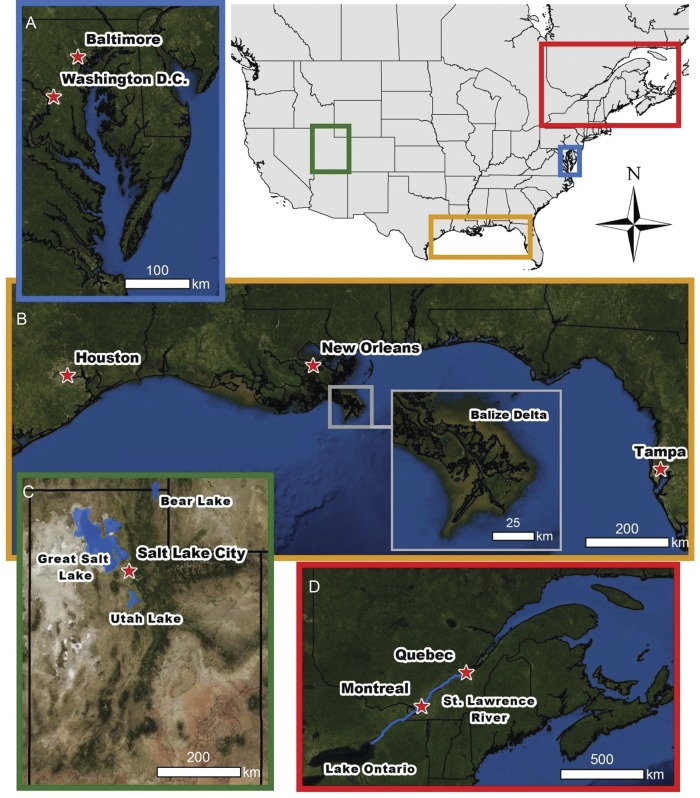
The locations of the four study regions in North America and more detailed images of (A) the Chesapeake Bay, (B) the Gulf Coast, (C) Utah and (D) the St Lawrence River.

Here we focus on the invasion of introduced *P. australis* in four regions in North America—the Chesapeake Bay, the St Lawrence River, Utah and the Gulf Coast (Fig. 1)—with distinctive climates, invasion histories, invasion characteristics and other resident *P. australis* lineages. For each region, we ask a number of questions related to invasion patterns, drivers and impacts: (i) What is the regional and continental significance of the region's wetlands? (ii) What are the known or perceived negative or positive impacts of *P. australis* invasion? (iii) In what habitats do you find the different *P. australis* lineages? (iv) When and how did introduced *P. australis* first invade and spread? (v) How fast is introduced *P. australis* expanding within the region? (vi) Is there evidence for multiple introductions of *P. australis*? (vii) Do the mechanisms of spread differ among the lineages? (viii) Is introduced *P. australis* replacing the other lineages? (ix) What are the major vegetation types that *P. australis* is replacing? We answer these questions by surveying the primary literature and unpublished data sets from each region. Note that we do not summarize findings related to hybridization between these lineages and sublineages; such information has been thoroughly reviewed in another article in this special issue (see Meyerson *et al.* 2012).

## Results – four regional case studies

The following descriptions of *P. australis* status in each of the four focus regions are summarized as answers to our nine questions in Table 2.

**Table 2:** The research questions addressed and summarized findings regarding the three *Phragmites* lineages in the four study regions.

	Chesapeake Bay	St Lawrence River	Utah	Gulf Coast
Regional climate (**www.worldclimate.com**; data from nearby meteorological stations)	Jan max/min = 5 °C/−5 °C; July max/min = 31 °C/19 °C; Average rainfall = 105 cm; Baltimore, MD	Jan max/min = −6 °C/−14.7 °C; Julymax/min = 26 °C/16 °C; Average rainfall = 76 cm; Montreal, Trudeau Airport, QC	Jan max/min = 3 °C/−7 °C; July max/min = 34 °C/17 °C; Average rainfall = 100–200 cm; Salt Lake City, UT; multiple nearby alpine sites for precipitation	Jan. max/min = 16 °C/6 °C; July max/min = 32 °C/23 °C; Average rainfall = 158 cm; Plaquemines Parish, LA
1. What is the regional and continental significance of the region's wetlands?	Fisheries including blue crab; migratory (Atlantic Flyway) and resident bird habitat	Drinking water; habitat for birds, fish, and other wildlife including many at-risk species	Critical migratory bird habitat on Pacific and Central Flyway, particularly for a region with a semiarid environment where wetlands are scarce	LA contains 40–45 % of the US's wetland habitats; an important stopover for birds on the Mississippi Flyway
2. What are the known or perceived negative or positive impacts of *P. australis* invasion?	Negative impact: Aggressive spread; recent study documenting no native plant diversity in *P. australis* stands Positive impact: May act as a sediment trap, buffering wetlands from sea-level rise	Negative impact: Large monospecific stands raise concerns about consequences for ecosystem function and wildlife habitat Positive impact: May be efficient at removing nutrients from agricultural run-off in ditches	Negative impact: Perceived but not documented loss of diverse habitat for migratory birds Positive impact: Unknown	Negative impact: Can negatively affect bird habitats; with progressive invasion of interior marshes, may cause loss of wildlife habitat Positive impact: Can help prevent marsh subsidence by capturing sediment and protecting interior marshes from tropical storm events and oil spills
3. In what habitats do you find the different *P. australis* lineages?	Introduced: fresh to brackish wetlands; associated with developed and agricultural land-use Native: rivers and creeks on eastern shore of Chesapeake Bay	Introduced: ditches; newly exposed shores; and managed, disturbed or restored wetlands Native: freshwater wetlands of the St Lawrence River (low marsh and areas with fewer human impacts)	Introduced: fresh to brackish wetlands; on sandy beaches, in seasonally flooded areas, and in semi-permanently flooded wetlands with emergent vegetation; disturbed habitats such as ditches and roadsides Native: along rivers and streams, in seeps or near hot springs, and usually away from the major lakes and cities; widespread but not dense	Introduced: mostly in Balize Delta, where it is the dominant vegetation in wetlands with depths <1 m *Berlandieri*: on roadsides, waste areas/lowlands adjacent to estuarine wetlands, and most wet soils in general, but usually not in standing water; in Balize Delta - sporadically on spoil banks and elevated splays
4. When and how did introduced *P. australis* first invade and spread?	Little documentation except rapid spread shown in Rhode River 1970–present day	Present for more than 96 years but spread rapidly with the creation of new habitat associated with the highway network in 1960–70s	First herbarium record in 1993; spread rapidly post-flooding of Great Salt Lake in 1980s	Introduced >90 years ago; arrival and spread likely related to major storm events and anthropogenic impacts from canal construction and dredging
5. How fast is introduced *P. australis* expanding within the region?	Number of patches in Rhode River increased 40× and area covered increased 25× over a 40-year period	Mean dispersal events for the establishment of new patches estimated at 27–77 m year^−1^ in linear habitats (roadside and agricultural ditches)	No published data	No published data. Ongoing studies looking at annual growth and spread of individual clones with different water depths and salinity levels
6. Is there evidence for multiple introductions of *P. australis*?	Most likely explanation given the high levels of genetic diversity	Most likely explanation given the high levels of genetic diversity	Most likely explanation given the high levels of genetic diversity	Yes, because there are multiple sublineages of introduced *P. australis*
7. Do the mechanisms of spread differ among the lineages?	Introduced: seeds very important within and between watersheds, and even within patches Native: no data	Introduced: seeds more important than previously thought Native: no data	Seeds much more important for introduced than native	Introduced: reliance on sexual reproduction varies between sublineages *Berlandieri* spread is almost entirely vegetative
8. Is introduced *P. australis* replacing the other lineages?	Not documented; co-occur only in some areas	Possibly at regional scale based on herbarium specimens; however monitoring at the boundary between native and introduced patches did not show clear replacement of one by the other	Not documented but historic native populations were found to still exist in a recent field survey; co-occur in a number of locations so native may get replaced in the near future	Not documented in the Balize delta (*berlandieri* does not play a significant role in deltaic wetlands); in competition study in a restoration, *berlandieri* was replaced by introduced *P. australis*
9. What are the major vegetation types that *P. australis* is replacing?	*Iva frutescens* (marsh elder), *Spartina patens* (salt meadow cordgrass), *Spartina cynosuroides* (big cordgrass), *Schoenoplectus americanus* (common threesquare), *Distichlis spicata* (saltgrass), and *Typha angustifolia* (narrowleaf cattail)	Has been associated with habitats supporting species such as *Typha spp.* (cattails)*, Carex lacustris* (hairy sedge)*, Sparganium eurycarpum* (broadfruit bur-reed)*,* and *Calamagrostis canadensis* (bluejoint). Introduced *P. australis* outcompetes *Typha* spp. in roadside habitats but no other systematic documentation of vegetation replacement	*Schoenoplectus maritimus* (alkali bulrush), *S. acutus* (hardstem bulrush), *Typha* spp. (cattails), and mudflat species such as *Distichlis spicata* (saltgrass)	In interior marshes of the Balize Delta, *Schoenoplectus deltarum* (delta bulrush), *Sagittaria latifolia* (broadleaf arrowhead), and *Sagittaria platyphylla* (delta arrowhead) are being invaded by introduced *P. australis*; but overall, there is a lack of historical data

### Native and introduced *P. australis* in the Chesapeake Bay

We begin with a synopsis of native and introduced *P. australis* in the Chesapeake Bay, since many of the ideas that have influenced the study of *P. australis* invasion in North America come from the eastern seaboard of the USA (e.g. Wijte and Gallagher 1996*a*, *b*; Chambers *et al.* 1998; Farnsworth and Meyerson 1999; Meyerson *et al.* 1999; Amsberry *et al.* 2000; Bart and Hartman 2000). The Chesapeake Bay is the largest estuary in North America, with a 26 000-ha watershed spanning six states and the District of Columbia. The climate is temperate, with hot and humid summers and relatively mild winters (Alliance for the Chesapeake Bay 2004). The wetlands of the Chesapeake Bay provide critical habitat—as a nursery, for feeding, and for cover—for the ∼200 species of fish that occur in the Bay (Metzgar 1973; Tiner and Burke 1995). In addition, the wetlands of the Chesapeake Bay are extremely important to migratory birds on the Atlantic Flyway; one-third of these birds actually winter in the Bay (Tiner and Burke 1995). The Chesapeake Bay is also world renowned for its blue crab (*Callinectes sapidus*), the Bay's most valuable fishery (**http://chesapeakebay.noaa.gov/fish-facts/blue-crab**). Blue crabs use most aquatic habitats of the Bay, including intertidal wetlands where *P. australis* often dominates, at some point during their life cycle.

The consequences of *P. australis* invasion and wholesale conversion of multi-species wetlands to monocultures of *P. australis* is an active area of research. In the Chesapeake Bay, introduced *P. australis* is typically considered undesirable because of its aggressive spread, and because of known negative effects of *P. australis* on diversity and ecosystem processes. In fact, a recent study in the Rhode River subestuary of the Chesapeake Bay documents that brackish tidal wetlands that have become dominated by introduced *P. australis* support few native plant species (M. Sievers, Smithsonian Environmental Research Center, unpubl. data). However, in some cases *P. australis*-dominated tidal wetlands might provide valuable services by acting as a sediment trap and thus buffering wetlands from sea-level rise (Rice *et al.* 2000). Although in other regions introduced *P. australis* may serve an important role in nutrient removal, there has been little research on such beneficial effects in nutrient-rich Chesapeake Bay wetlands.

**Fig. 2:**
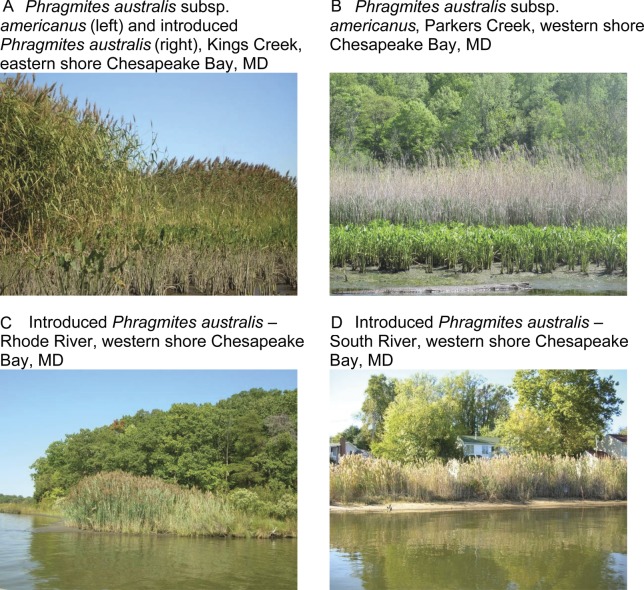
**The two *P. australis* lineages—subspecies *americanus* and introduced—in the Chesapeake Bay**. Photographs (A), (C) and (D) by K. Kettenring, and (B) by D. F. Whigham.

Introduced *P. australis*, most likely the Short B sublineage (based on earlier surveys; see analysis in Hauber *et al.* 2011), is found throughout the Chesapeake Bay in fresh to brackish wetlands (Fig. 2). A field survey by Chambers *et al*. (2008) revealed that *P. australis* was found along 15 % of surveyed estuarine shoreline in Maryland and 2 % in Virginia, and was often associated with agricultural shoreline. King *et al*. (2007) found that introduced *P. australis* was more abundant in subestuaries of the Chesapeake that had watersheds dominated by anthropogenic development as opposed to forested watersheds. Subestuaries with developed watersheds also had higher nitrogen levels in their water, and the *P. australis* in those subestuaries had higher foliar nitrogen levels. Taken together, these two studies suggest an important role of human disturbance and nutrient enrichment in *P. australis* invasion in the Chesapeake Bay. Native *P. australis* is found in rivers and creeks throughout the eastern shore of Maryland, particularly in the Nanticoke and Choptank rivers (Meadows and Saltonstall 2007).

The only information concerning the initial invasion of introduced *P. australis* in the Chesapeake Bay is that it was present in the early 1900s at Chesapeake Beach, MD (Saltonstall 2002; Saltonstall *et al.* 2004). More recently, a detailed study by McCormick *et al*. (2010*a*) of introduced *P. australis* spread in the Rhode River subestuary of the Chesapeake Bay found that over a 40-year period (1970–2007), the number of *P. australis* patches increased from 5 to 212 and the area occupied by *P. australis* increased from 0.73 to 18 ha. Another study documented high intrinsic rates of increase of *P. australis* patches in the Chesapeake Bay (0.06–0.19 year^−1^ in more recently colonized brackish wetlands), but it is not clear as to whether patches that were followed were native or introduced (Rice *et al.* 2000). While there is no direct evidence for multiple introductions of *P. australis* in Chesapeake Bay wetlands, McCormick *et al*. (2010*a*, *b*) found substantial genetic diversity in *P. australis* populations, supporting the notion that there have been multiple introductions.

Genetic analyses of introduced *P. australis* populations across nine subestuaries of the Chesapeake Bay and detailed study of all *P. australis* patches within the Rhode River subestuary indicate that seeds are the predominant means of movement within and between subestuaries (McCormick *et al.* 2010*a*, *b*), while spread within patches appears to be a mixture of clonal and seed propagation. The ability of introduced *P. australis* patches to spread by seed can vary because: (i) seed viability differs greatly between patches, due to the availability of out-crossed pollen, (ii) *P. australis* seed densities in seed banks reflect patch-level viable seed production, and (iii) floret and inflorescence production is driven strongly by nutrient levels (Kettenring and Whigham 2009; Baldwin *et al.* 2010; Kettenring *et al.* 2010, 2011). Each of these factors drives variability in reproductive output and potential spread by seed between introduced *P. australis* patches. How these mechanisms of spread compare to native *P. australis* is not known.

Introduced *P. australis* has colonized both tidal freshwater and brackish wetlands in the Chesapeake Bay, over a wide range of salinities. In Maryland tidal wetlands, *P. australis* appears to be able to invade all plant communities, eventually displacing species such as *Iva frutescens*, *Spartina patens*, *Spartina cynosuroides*, *Schoenoplectus americanus*, *Distichlis spicata* and *Typha angustifolia* (D. Whigham, Smithsonian Environmental Research Center, pers. comm.). However, sometimes these species and others (e.g. *Smilax rotundifolia*, *Apios americana* and *Acer rubrum* on the forest border; M. Sievers, unpubl. data) are able to persist in the leading edges of developing *P. australis* patches. It is likely that there is some replacement of native *P. australis* by introduced *P. australis* in the habitats where they co-occur, but there have not been systematic studies to document this phenomenon.

### *Phragmites australis* in the St Lawrence river system

**Fig. 3:**
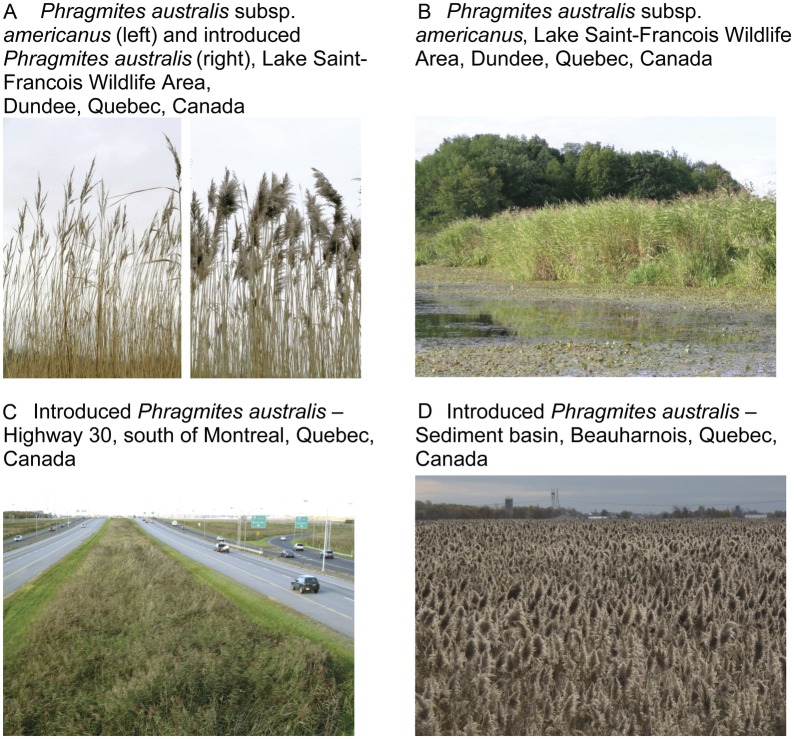
**The two *P. australis* lineages—subspecies *americanus* and introduced—in the St Lawrence River region**. Photographs by J. Brisson.

In the St Lawrence River system of Canada, invasion by *P. australis* has been widely reported predominantly in non-tidal freshwater wetlands and in newly created anthropogenic habitats such as roadsides and agricultural ditches (Fig. 3; Gervais *et al.* 1993; Lavoie *et al.* 2003; Wilcox *et al.* 2003; Hudon *et al.* 2005; Maheu-Giroux and de Blois 2007; Jodoin *et al.* 2008); this invasion is most likely by the Short B sublineage (see the analysis in Hauber *et al.* 2011). The invasion of linear habitats along roadsides has been especially spectacular in the last few decades and has resulted in a vast network of well-connected populations (Brisson *et al.* 2010).

The St Lawrence River drains the world's largest system of freshwater lakes and is the third-largest drainage basin in North America. The climate is continental, with hot, humid summers and cold, snowy winters. The freshwater wetlands along its major fluvial lakes and banks harbour a large proportion of Canada's at-risk species (Environment Canada 2010), and several of the basin's national wildlife areas and migratory bird sanctuaries are recognized as internationally significant. Whereas the rate of wetland loss has declined in recent years along parts of the fluvial corridor (Jean and Létourneau 2011), scientists believe that most of the original wetlands of the St Lawrence valley may have already disappeared. Over 50 exotic plant species have colonized the remaining wetlands, with introduced *P. australis* being among the most conspicuous. The proportion of plant cover occupied by exotic species tends to be higher in the densely populated fluvial sectors of the St Lawrence than in the estuarine portions (Lavoie *et al.* 2003).

Three situations in particular have contributed to rising concerns over the environmental impact of introduced *P. australis.* First, the construction of new transport infrastructures and the drainage of lowlands for agriculture in the St Lawrence valley in the 1960s and 1970s were followed by the rapid and very conspicuous *P. australis* invasion of linear wetlands associated with these infrastructures (roadside and agricultural drainage ditches) (Maheu-Giroux and de Blois 2005, 2007; Jodoin *et al.* 2008). The proliferation of a well-connected invasion network can lead to increased *P. australis* propagule pressure on natural wetlands (Taddeo and de Blois 2012), but in an agricultural context with heavy nutrient loading *P. australis* may also be beneficial as an effective barrier that filters water and traps sediment. Second, episodes of low water levels in the St Lawrence River system as a result of climatic fluctuations led to the massive invasion of shores by introduced *P. australis* (Hudon *et al.* 2005), raising concerns over its impacts on fish spawning sites and waterfowl. Finally, numerous wetland restoration sites have become dominated by introduced *P. australis*, with potential loss of quality habitat for the species these wetlands were intended to protect (A. Michaud, Ducks Unlimited, pers. comm.). In all these cases, the invasion of *P. australis* is viewed as a consequence of management practices that resulted in ideal conditions for this opportunistic species.

Evidently, introduced *P. australis* is highly competitive on disturbed sites and new anthropogenic habitats, and this is nowhere better exemplified than in roadside habitats of the St Lawrence valley. Genetic analyses of >260 leaf samples obtained from an intensive survey along 1359 1-km highway sections in Quebec showed that they were overwhelmingly introduced *P. australis*. The native lineage, on the other hand*,* is mostly found along the last remaining large freshwater wetlands of the St Lawrence River (Lake Saint-François, Lake Saint-Louis, Lake Saint-Pierre; Jodoin *et al.* 2008). A detailed study of *P. australis—*in one of these large remaining freshwater wetlands where the two types co-occur—shows that the introduced and native lineages are associated with distinct land uses and land covers (Taddeo and de Blois 2012). Native *P. australis* is mostly found in low marshes and areas with fewer human impacts, whereas introduced *P. australis* is associated with roads (or other disturbances) and drier areas. These patterns are similar to those found at the regional scale for introduced and native *P. australis* and suggest that co-existence is possible, at least for some time.

To reconstruct the historical spatial distribution of *P. australis* at the regional scale, Lelong *et al*. (2007) mapped the location of herbarium specimens from Quebec through time and conducted genetic analyses on the specimens in historical collections. The oldest native specimen was collected on the shore of a river in the far east of the study area in 1882, whereas the oldest available introduced specimen was collected in 1916 along the St Lawrence River southeast of Quebec City. Introduced *P. australis* has thus been present for at least 96 years in this region and may have been introduced by boats through exchanges with Europe well before that. Multiple introductions are likely, given the high level of genetic diversity among populations of introduced *P. australis* (Belzile *et al.* 2010; Kirk *et al.* 2011).

In herbarium collections, most of the specimens collected prior to the 1970s are of native *P. australis*; the vast majority of the specimens collected or sampled after that point are of the introduced lineage. The 1960s and 1970s were a period of historically low water levels in the St Lawrence River, and coincided with the expansion of the Quebec highway system and agricultural intensification. Focusing on linear wetlands (roadside and agricultural ditches, riparian habitats), Maheu-Giroux and de Blois (2005) used aerial photographs to reconstruct the spread from the 1980s to 2002 of introduced *P. australis* in peri-urban landscapes. Very high rates of increase were observed, with populations more than doubling in spatial extent each year. Interestingly, riparian habitats, being less disturbed, were also less invaded. New populations established on average 27–77 m away from alreadyestablished patches, although rare longer-distance dispersal events also occurred. Densification of the patches was facilitated by nutrient-rich agricultural run-off in a time of rapid intensification of agricultural activities.

Given that clonal propagation is usually vigorous, the contribution of sexual reproduction to the spread of introduced *P. australis* has been somewhat overlooked until recently. Three lines of evidence have been used to assess invasion mechanisms. First, the numerous colonization events and dispersal patterns observed by Maheu-Giroux and de Blois (2007) within and between linear habitats suggested a more important role of seed dispersal than previously acknowledged. Low seed germination rates are compensated for, to some extent, by very high seed production rates. Second, Brisson *et al*. (2008) directly observed seedling establishment and survival over two growing seasons in roadside ditches. They suggested that warming in recent years may have contributed to increased seed production and seedling survival for the introduced lineage, a hypothesis that is currently being investigated. Finally, genetic studies have reported high rates of genetic diversity among populations of introduced *P. australis* at both the landscape (Belzile *et al.* 2010) and regional scale (Kirk *et al.* 2011), with both long-distance and short-distance dispersal events determining population structure (Maheu-Giroux and de Blois 2007; Kirk *et al.* 2011). It is possible that the contribution of sexual reproduction has increased with time with increased density of populations and the availability of out-cross pollen (Kettenring *et al.* 2010, 2011).

Unlike the introduced lineage, the native lineage is not known to progress regionally by colonizing previously unoccupied wetland sites. In sites where the introduced and native lineages co-occur, however, populations of both types can expand locally mostly by clonal propagation, with a non-significant trend towards faster densification of the introduced lineage (S. de Blois, unpubl. data). Direct pre- and post-invasion surveys at the edge of an invasion front would be needed to evaluate the effect of the spread of the introduced lineage on resident plant communities. This would help clarify whether low plant diversity patterns reported in invaded systems result from the competitive effect of *P. australis* on resident plants or the fact that already species-poor disturbed habitats were invaded. Disturbed habitats are easily invaded, but if seeds of *P. australis* reach a resident plant community, that community will offer resistance to *P. australis* seedling establishment, and the level of invasion resistance will depend on the plant functional groups present (Byun *et al.* 2013).

At the regional scale, historical herbarium records suggested that native *P. australis* were being replaced by the introduced lineage over time (Lelong *et al.* 2007). S. de Blois and colleagues (unpubl. data) monitored five locations with native and introduced populations, for periods ranging up to 5 years, to verify the displacement hypothesis. They found that competitive outcomes can vary with site conditions, but native *P. australis* resisted invasion better than expected. Landscape distribution patterns also show that native *P. australis* occupies less-disturbed habitats, where it may manage to escape competition (Taddeo and de Blois 2012).

In the freshwater and brackish portions of the St Lawrence River, introduced *P. australis* has been associated with habitats supporting species such as *Typha* spp., *Carex lacustris*, *Sparganium eurycarpum* and *Calamagrostis canadensis*, as well as with other introduced species such as *Lythrum salicaria* and *Phalaris arundinacea*. Introduced *P. australis* has been shown to outcompete *Typha* spp. for space in roadside habitats and wetlands (Bellavance and Brisson 2010), but as yet there is no direct report of the replacement of other vegetation types by introduced *P. australis*. Comparisons of fish, bird and amphibian populations in invaded and non-invaded wetland habitats have found limited support for negative impacts of introduced *P. australis* on fauna (Le Groupe Phragmites 2012).

### Native and introduced *P. australis* in Utah

The introduction of *P. australis* in Utah appears to be the most recent invasion relative to the other regions of North America reported here, but given its progression in wetlands and other habitats, it is likely to become as significant a factor as in the other regions if left unchecked. Most wetlands in Utah are found in the northern part of the state in the Great Salt Lake watershed, which includes the wetlands around Bear Lake and Utah Lake, and riparian and other wetlands throughout the watershed (Fig. 1). Salt Lake City, the major metropolitan area in the Wasatch Front, the area east of the Great Salt Lake, has a semi-arid climate characterized by warm, dry summers and cold, snowy winters. Most wetlands in northern Utah are fed largely by snow melt and are critical habitat for wildlife including migratory waterfowl and shorebirds on the Pacific and Central Flyways (Evans and Martinson 2008). In fact, the Great Salt Lake and its 160 000 ha of wetlands have been designated a Western Hemisphere Shorebird Reserve Network site with a ‘hemispheric rank’ (Western Hemisphere Shorebird Reserve Network 2009). Perhaps the most important wetlands on the Great Salt Lake are found in the Bear River Migratory Bird Refuge, where the 1000 km Bear River terminates on the northeast arm of the Great Salt Lake. These 29 000 ha of wetlands, playas and mudflats are used by >260 species of birds, including 33 species of shorebirds (Olson *et al.* 2004; Denton 2007). During the fall migration as many as 500 000 ducks and 200 000 shorebirds visit the Refuge (Olson *et al.* 2004).

The invasion of introduced *P. australis* (presumably Short B; see analysis in Hauber *et al.* 2011) into wetlands in Utah, particularly in the Great Salt Lake watershed, is perceived as one of the biggest threats to the state's native wetland plant diversity and wildlife habitat quality (Olson 2007; Kettenring and Mock 2012). For that reason, there are major efforts on the part of private, state and federal land managers to control *P. australis* in Utah's wetlands. Documentation of the impacts of *P. australis* invasion, however, is lacking in this region.

**Fig. 4:**
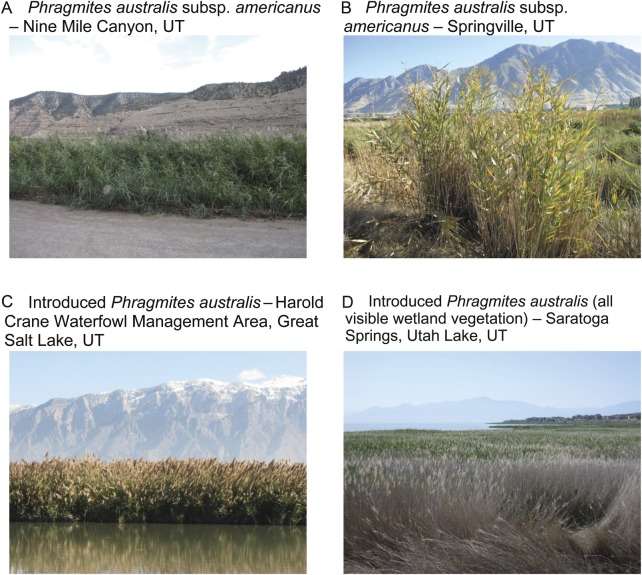
**The two *P. australis* lineages—subspecies *americanus* and introduced—in Utah**. Photographs by K. Kettenring.

Introduced *P. australis* is found predominantly in the northern half of the state, in the Great Salt Lake watershed in the corridor from Bear Lake to Utah Lake (Meyerson *et al.* 2010; Kulmatiski *et al.* 2011; Kettenring and Mock 2012). Introduced *P. australis* occurs in fresh to brackish wetlands, on sandy beaches, in seasonally flooded areas, and in semi-permanently flooded wetlands with emergent vegetation (Fig. 4). Introduced *P. australis* is also a dominant feature of highly disturbed habitats such as roadsides and ditches. On the other hand, subsp. *americanus* occurs throughout the state, including central and southern Utah, in freshwater habitats along rivers and streams, in seeps or near hot springs, and usually away from the major lakes and cities. There are, however, a few populations of native *P. australis* along each of the three major lakes (K. M. Kettenring, pers. observ.; Kulmatiski *et al.* 2011; Kettenring and Mock 2012).

Many wetland managers think *P. australis* began to spread rapidly after major floods of the Great Salt Lake in the 1980s (Kettenring *et al.* 2012). (Such floods occur periodically as natural fluctuations on a decadal scale.) When the floodwaters receded through the latter part of the decade and into the 1990s, they left vast areas of mudflats. Interestingly, these anecdotes about the timing of rapid *P. australis* invasion are confirmed by recent analyses which show that the earliest recorded herbarium specimen of introduced *P. australis* was from 1993, from near Camp Williams (between the Great Salt Lake and Utah Lake, ∼40 km south of Salt Lake City; Kulmatiski *et al.* 2011). The rates of expansion have not been calculated for introduced *P. australis*, but clearly it has spread rapidly in <20 years to become a dominant feature in northern Utah wetlands. The extent to which human dispersal plays a role in *P. australis* invasion has not been determined, although it appears that the major corridor of invasion has been along Interstate 15 in the northern part of the state.

A recent study by Kettenring and Mock (2012) suggests that spread by seed is much more important for introduced *P. australis* than for subsp. *americanus* at the landscape scale in Utah. Within patches, introduced *P. australis* spreads both by clonal propagation and by seeds, depending on the site, while for subsp. *americanus*, most spread is clonal. The substantial amount of genetic variation Kettenring and Mock (2012) found in introduced *P. australis* suggests that multiple introductions have occurred, but this hypothesis has not been explicitly tested.

There is no evidence that introduced *P. australis* has yet widely displaced subsp. *americanus*. Kettenring and Mock (2012) were able to identify native *P. australis* in many of the historic herbarium collection locations described in Kulmatiski *et al*. (2011). Still, there are a number of places where introduced *P. australis* and subsp. *americanus* co-occur that should be closely monitored. These include sites on Utah Lake, in Cutler Marsh in Cache Valley (which is between Bear Lake and Great Salt Lake), on the north side of Bear Lake, and on the north side of the Great Salt Lake (Fig. 1; K. M. Kettenring, pers. observ.). Although there have not been detailed studies of the type of vegetation that introduced *P. australis* is replacing, field observations indicate that it takes over areas occupied by other emergent wetland plants such as *Schoenoplectus maritimus*, *S. acutus*, *Typha* spp., and mudflat species such as *Distichlis spicata* (Olson 2007; K. M. Kettenring, pers. observ.).

### Introduced *P. australis* and subsp. *berlandieri* in the Gulf Coast

**Fig. 5:**
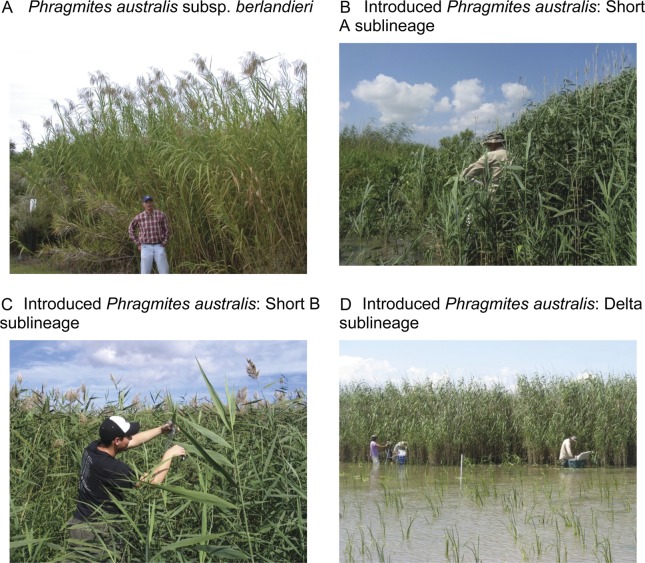
**The two *P. australis* lineages—subspecies *berlandieri* and introduced—and three of the introduced sublineages in the Gulf Coast**. Photographs by C. S. Hood.

A significant aspect of the introduction of *P. australis* in the Gulf Coast region is the level of genetic variation present, specifically in the Mississippi River Balize delta (Figs 1 and 5). Whereas in the other regions of North America reported here, introduced *P. australis* is believed to consist primarily of a single sublineage (Short B; see analysis in Hauber *et al.* 2011), the introduced lineage in the Balize delta consists of possibly five sublineages: Short A, Short B, Delta, Greeny 2 (Hauber *et al.* 2011; Lambertini *et al.* 2012), and Greeny 3 (one sample; Lambertini *et al.* 2012) (Table 1). Short A and Greeny 2 exhibit a similar morphology in that populations have a distinct blue–green colour, are ≤2.5 m at maturity, and begin flowering in early summer (Lambertini *et al.* 2012; D. A. White, D. P. Hauber and C. S. Hood, pers. observ.). Short B is similar in height and flowering time, but lacks the distinctive blue–green colour. Delta, the predominant sublineage in the Balize delta, is much taller at maturity (≥3 m) and begins flowering in late October (Hauber *et al.* 2011). Delta thrives at water depths ≤1 m, although it tolerates slightly deeper water if already established and it does not spread under these conditions. It easily tolerates periods of elevated salinity during tropical storm events. With the presence of these different sublineages, the Balize delta is a unique crucible for studying novel genetic recombinations, ecological and phenotypic variation, and intraspecific interactions.

The northern Gulf of Mexico coastline has a subtropical climate, with hot, humid summers and mild winters (**www.worldclimate.com**). Wetlands are the dominant habitats in this region. Louisiana alone contains 40–45 % of the wetlands of the continental US, and most occur as coastal marshes (as defined by Keddy 2000). The northern Gulf of Mexico contains 58 % of coastal marshes of the continental US (Alexander *et al.* 1986), most of which are the result of deltaic deposits of the meandering mouth of the Mississippi River over the past 4000 years (Gosselink and Baumann 1980). The northern US Gulf Coast marshes serve as nurseries supporting the production of >30 % of domestic seafood in the Gulf of Mexico (Chabreck 1988; Chesney *et al.* 2000). The quality habitat required by these fisheries is jeopardized by wetland loss caused by anthropogenic factors, subsidence, coastal erosion and sea-level rise.

In the diverse interior marshes of the Balize delta, introduced *P. australis* is seen as having both negative and positive impacts. It decreases the foraging habitat for migratory birds on the Mississippi Flyway, which do not feed on *P. australis*. In these interior marshes, *Schoenoplectus deltarum*, *Sagittaria latifolia* and *Sagittaria platyphylla* are being replaced by both *Typha* spp. and introduced *P. australis* (D. A. White, pers. observ.). On the other hand, in the outer delta marshes where introduced *P. australis* is by far the dominant emergent plant species, wetland managers see it as a benefit due to its capacity to trap sediment, allowing for stabilization from subsidence. It also serves to buffer and protect the diverse interior marshes, particularly during tropical storm events. Introduced *P. australis* is salt tolerant. It also tolerates other disturbances: following the British Petroleum oil spill in 2010, though the oil-covered *P. australis* culms died off, many of the stands appeared to be producing new shoots from perennial growth below the waterline (D. A. White, pers. comm.), thus buffering interior marshes from the effects of the spill.

It has been estimated (as described in Hauber *et al.* 2011) that the initial introduction of the Delta sublineage in the Balize delta was ∼90 years ago, based on historical accounts and the fact that the main river distributaries in the Balize delta have experienced over 200 years of international passages along with periodically spilled cargo and dumped ballast water. Over time, periodic tropical storm events and anthropogenic impacts via canal construction and channel dredging are believed to have accelerated subsidence and likely provided greater opportunity for introduced *P. australis* to spread. The Delta sublineage's ancestral origin appears to be from a Mediterranean/African population (Hauber *et al.* 2011; Lambertini *et al.* 2012). The other four introduced sublineages appear to be very recent, likely occurring within the past 20 years (Hauber *et al.* 2011).

The other lineage of *P. australis* found in this region, subsp. *berlandieri*, is likely the resident taxon on the Gulf Coast and possibly has dispersed there naturally from populations in Central and South America, which are more diverse (Lambertini *et al.* 2012; D. P. Hauber, unpubl. data). Based on observations from field studies along the Gulf Coast over the past 25 years, subsp. *berlandieri* lacks aggressive growth (D. P. Hauber and D. A. White, pers. observ.). It commonly occurs on roadsides, lowlands and wet soils in general, but usually not in standing water (Fig. 5). In the Balize delta, it occurs sporadically on spoil banks and elevated splays (D. P. Hauber, D. A. White and C. S. Hood, pers. observ.).

The different *P. australis* lineages and sublineages along the Gulf Coast do seem to vary in their primary mechanism of propagation. Subspecies *berlandieri* (6× ploidy; Gaudreault *et al.* 1989; Hauber *et al.* 2011) appears to spread entirely clonally given its low genetic diversity based on microsatellite analysis and lack of viable seed based on germination trials (D. P. Hauber, unpubl. data). Delta, Short B and Greeny 2 have displayed inter-population genetic variation and/or viable seed production, indicating that sexual propagation is playing at least a minor role in their spread. However, over half of the Delta sublineage populations sampled shared the same multilocus allele (nuclear) phenotype, and similarly for the Short A sublineage, so undoubtedly clonal spread is the primary mechanism for most of the introduced sublineages (Hauber *et al.* 2011). Preliminary data from growth rate studies of select clones in the Balize delta show that all introduced sublineages seem to exhibit rapid clonal spread (D. A. White, D. P. Hauber and C. S. Hood, unpubl. data).

Without archived material older than 30 years, it is not possible to say if replacement of the ‘resident’ subsp. *berlandieri* by Delta has occurred in introduced *P. australis*-dominated exterior marshes of the Balize delta. It is clear that in those locations subsp. *berlandieri* is infrequent, and colonies exhibit slow clonal spread (D. A. White, D. P. Hauber and C. S. Hood, unpubl. data). In the past 10 years, introduced *P. australis* along with *Typha* spp. have successfully invaded some interior marshes, converting large portions of the diverse vegetation from a dominant *Schoenoplectus deltarum* and *Sagittaria* spp. marsh to mostly introduced *P. australis* and *Typha* spp. (D. A. White, pers. comm.). Also, competition studies conducted in the Barataria marshes of Louisiana indicate that the introduced *P. australis* lineage easily outcompetes subsp. *berlandieri* as well as *Schoenoplectus* spp. and *Distichlis spicata* (Howard *et al.* 2008).

## Discussion

Our regional assessments reveal substantial ecological diversity between and within the lineages of *P. australis*. These observations are supported by recent genetic advances. We now see that the variation within the species reflects more complexity than previously considered. The introduced lineage alone occurs in diverse climates, in wetland types with widely varying environmental conditions and disturbance regimes, and exhibits differing reproductive strategies. It must still be determined whether these variations reflect inherent differences in ecological requirements, or are evidence for extreme plasticity within a species and adaptations to local conditions; or if these different sublineages are representative of a cryptic species complex within introduced *P. australis*, as demonstrated by recent studies in the Balize delta. As researchers discover more sublineages of introduced *P. australis*, collaboration at the continental level will be important in identifying patterns of genetic diversity that may be associated with different invasion patterns. At a broad level, comparisons between the introduced and native lineages have clearly shown ecological and phenological differences, but more research is required to identify the contribution of genetic diversity to ecological differences within the introduced lineage, or the native lineage for that matter. In regions other than the Gulf Coast, Short B appears to be the predominant invasive sublineage, but as Delta or other introduced sublineages spread more widely, careful identification will be important.

Comparing the four regions, we found interesting and surprising differences within the introduced lineage in terms of the timing of introduction, the habitat occupied and mode of reproduction. In Utah and the Chesapeake Bay, introduced *P. australis* is found in a diversity of wetlands and newly created habitats, while in the St Lawrence and Gulf Coast, the preferred habitat is narrower. Also, although sexual reproduction is common for introduced *P. australis* in Utah, the Chesapeake Bay and the St Lawrence River, in the Gulf Coast there is substantial variation in mode of reproduction in the introduced sublineages. Mode of reproduction and adaptation to different environments can drive the timing and pattern of invasion in *P. australis* (Kettenring and Mock 2012), thus gaining a better understanding of what is driving these differences is critical.

Despite differences within the introduced lineage, we do see an omnipresent characteristic in *P. australis* invasion across North America: this lineage is highly opportunistic. It has been shown to invade disturbed or newly created habitats in the four study regions, areas where there is little to no competition from other vegetation types and likely high resource availability. Similar findings have been found in other regions of North America as well (e.g. Minchinton and Bertness 2003; Silliman and Bertness 2004). These initial disturbances seem to be important for establishment especially by seeds and may indicate ways of limiting the species in targeted locations. Once established, *P. australis* can quickly proliferate, even in pristine wetlands.

Looking to the future, it is important to consider what other opportunities humans may be providing for *P. australis* invasion. For instance, currently in the St Lawrence River watershed, large sections of the highway system, which are over 40 years old, are being rebuilt, with the resulting widespread disturbance of roadsides. Water levels are also at record lows, exposing suitable shoreline habitats. In Utah, human movement of propagules and human-caused disturbances may trigger the invasive lineage to become more widespread in the remote places where native *P. australis* is still dominant. In the Gulf Coast, introduced *P. australis* may become a dominant feature of interior marshes of the Balize delta, while in the Chesapeake Bay we may see further expansion into forested watersheds where the species is currently uncommon (King *et al.* 2007) as anthropogenic development increases. Even if the introduced lineage has been present for a long time in North America, conditions that favour the continental expansion of *P. australis* have increased in recent times, suggesting continued expansion in all regions.

In spite of some research on the biological impacts of introduced *P. australis* on wetland ecosystems (e.g. Meyerson *et al.* 1999; Keller 2000; Talley and Levin 2001; Able *et al.* 2003; Windham and Ehrenfeld 2003; Minchinton *et al.* 2006), a continental perspective highlights that there is insufficient or conflicting evidence regarding impacts on the flora and fauna. In the St Lawrence River, researchers have found more plant diversity in native vs. introduced stands of *P. australis* (S. de Blois, unpubl. data), but additional studies are required to determine if this is a result of an invasion effect or other factors such as differences in environmental conditions. At the current level of invasion, researchers have also observed no significant difference in the use by birds, fish or amphibians of introduced *P. australis* stands compared with adjacent non-invaded vegetation types (Le Groupe Phragmites 2012) but some habitat thresholds may have to be reached at the landscape scale before impacts on the fauna can be identified. Similarly, researchers following the invasion front of introduced *P. australis* into native *P. australis* stands have found limited evidence to indicate that native stands are being replaced rapidly by introduced *P. australis* (S. de Blois, unpubl. data). All these findings highlight the need for long-term monitoring that takes into account vegetation patterns and habitat diversity at the landscape scale, in order to better predict the competitive outcomes and impacts on biodiversity; and monitoring across regions using similar approaches to facilitate generalization.

Given that introduced *P. australis* is here to stay, it is important to reflect on both the positive and negative impacts of the sublineages. In the Mississippi River delta, introduced *P. australis* may be protecting inner natural marshes from storms and oil spills. If it invades interior marshes as well, however, it may negatively impact important wildlife habitat. In the St Lawrence, introduced *P. australis* may fill a role in new habitats along agricultural fields by processing the nutrient-rich run-off, but it may conflict with biodiversity conservation in adjacent species-rich wetlands or even with production on agricultural land. So, although *P. australis* has been widely perceived as a threat to wetland ecosystems, evidence of its impacts on biodiversity and ecosystem services still needs to be better documented. It should be recognized that the nature of these impacts is likely to change with time since invasion.

## Conclusions and forward look

Our comparison highlights important research priorities that can drive further efforts to inform a continental perspective. There is a definite need to better clarify the genetic and ecological relationships between the different introduced sublineages observed in North America, and their relative competitive ability and potential for admixture. This may be done through regional studies that use similar methodologies and share results to uncover common patterns and processes. To our knowledge, such studies have not been performed on *P. australis* in spite of the broad attention given to this species. A continental perspective can help untangle the relationships between the introduced sublineages, reproductive strategy and environmental changes such as nutrient enrichment or disturbances. Such research could advance theoretical knowledge on biological invasion by helping to determine the extent to which the patterns observed can be generalized or are sublineage specific or region specific. Controlled experiments, long-term monitoring and perhaps a functional approach across different ecological settings could be used to improve knowledge. When the regions are analysed in isolation from each other, invasion patterns sometimes appear idiosyncratic and resist generalization. It may be time to consider initiatives at the continental (if not intercontinental) scale to tackle unresolved issues.

## Sources of funding

K.K. acknowledges the UT Agricultural Experiment Station, the Gardner Junior Faculty travel award, and the UT State University Center for Women and Gender for funding. S.d.B. acknowledges Les Fonds de Recherche du Québec—Nature et technologies, and The Natural Sciences and Engineering Research Council of Canada for funding. D.H. thanks Loyola University Grants and Leaves Committee Research Grant, Faculty Development Grant, and Sabbatical Leave; Rev. J.H. Mullahy Fund; the Coastal Restoration and Enhancement through Science and Technology program; and the LA Board of Regents grant LEQSF(2007-12)-ENH-PKSFI-PES-03 to Frank Jordan.

## Contributions by the authors

All authors jointly wrote the manuscript.

## Conflict of interest statement

None declared.

## References

[PLS040C1] Able KW, Hagan SM, Brown SA (2003). Mechanisms of marsh habitat alteration due to *Phragmites*:
response of young-of-the-year mummichog (*Fundulus heteroclitus*) to
treatment for *Phragmites* removal. Estuaries and Coasts.

[PLS040C2] Alexander CE, Broutman MA, Field DW (1986). An inventory of coastal wetlands of the USA.

[PLS040C3] **Alliance for the Chesapeake Bay** (2004). Riparian forest buffers—linking land and water.

[PLS040C4] Amsberry L, Baker MA, Ewanchuk PJ, Bertness MD (2000). Clonal integration and the expansion of *Phragmites
australis*. Ecological Applications.

[PLS040C5] Baldwin AH, Kettenring KM, Whigham DF (2010). Seed banks of *Phragmites australis*-dominated brackish
wetlands: relationships to seed viability, inundation, and land cover. Aquatic Botany.

[PLS040C6] Bart D, Hartman JM (2000). Environmental determinants of *Phragmites australis*
expansion in a New Jersey salt marsh: an experimental approach. Oikos.

[PLS040C7] Bellavance M-E, Brisson J (2010). Spatial dynamics and morphological plasticity of common reed
(*Phragmites australis*) and cattails (*Typha* sp.) in
freshwater marshes and roadside ditches. Aquatic Botany.

[PLS040C8] Belzile F, Labbé J, LeBlanc M-C, Lavoie C (2010). Seeds contribute strongly to the spread of the invasive genotype of the
common reed (*Phragmites australis*). Biological Invasions.

[PLS040C9] Brisson J, Paradis É, Bellavance M-È (2008). Evidence of sexual reproduction in the invasive common reed
(*Phragmites australis* subsp. *australis*; Poaceae) in
eastern Canada: a possible consequence of global warming. Rhodora.

[PLS040C10] Brisson J, de Blois S, Lavoie C (2010). Roadside as invasion pathway for common reed (*Phragmites
australis*). Invasive Plant Science and Management.

[PLS040C11] Byun C, de Blois S, Brisson J (2013). Plant functional group identity and diversity determine biotic resistance
to invasion by an exotic grass. Journal of Ecology.

[PLS040C12] Chabreck RA (1988). Coastal marshes: ecology and wildlife management.

[PLS040C13] Chambers RM, Mozdzer TJ, Ambrose JC (1998). Effects of salinity and sulfide on the distribution of *Phragmites
australis* and *Spartina alterniflora* in a tidal
saltmarsh. Aquatic Botany.

[PLS040C14] Chambers RM, Havens KJ, Killeen S, Berman M (2008). Common reed *Phragmites australis* occurrence and adjacent
land use along estuarine shoreline in Chesapeake Bay. Wetlands.

[PLS040C15] Chesney EJ, Baltz DM, Thomas RG (2000). Louisiana estuarine and coastal fisheries and habitats: perspectives from a
fish's eye view. Ecological Applications.

[PLS040C16] Denton C (2007). Bear River: last chance to change course.

[PLS040C17] **Environment Canada** (2010). Species at Risk Act: Annual report for 2009. http://www.ec.gc.ca.

[PLS040C18] Evans K, Martinson W (2008). Utah's featured birds and viewing sites: a conservation platform for IBAs
and BHCAs.

[PLS040C19] Farnsworth EJ, Meyerson LA (1999). Species composition and inter-annual dynamics of a freshwater tidal plant
community following removal of the invasive grass, *Phragmites
australis*. Biological Invasions.

[PLS040C20] Galatowitsch SM, Anderson NO, Ascher PD (1999). Invasiveness in wetland plants in temperate North America. Wetlands.

[PLS040C21] Gaudreault SM, White DA, Hauber DP (1989). *Phragmites australis*: an analysis of reproductive
differences in two adjacent populations in the Mississippi River delta. American Journal of Botany.

[PLS040C22] Gervais C, Trahan R, Moreno D, Drolet A-M (1993). *Phragmites australis* in Quebec: geographical distribution,
chromosome number, and reproduction. Canadian Journal of Botany.

[PLS040C23] Gosselink JG, Baumann RH (1980). Wetland inventories: wetland loss along the United States
coast. Zeitschrift für Geomorphologie NF Suppl Bd.

[PLS040C24] Hauber D, Saltonstall K, White D, Hood C (2011). Genetic variation in the common reed, *Phragmites
australis*, in the Mississippi River delta marshes: evidence for multiple
introductions. Estuaries and Coasts.

[PLS040C25] Howard R, Travis S, Sikes B (2008). Rapid growth of a Eurasian haplotype of *Phragmites
australis* in a restored brackish marsh in Louisiana, USA. Biological Invasions.

[PLS040C26] Hudon C, Gagnon P, Jean M (2005). Hydrological factors controlling the spread of common reed
(*Phragmites australis*) in the St. Lawrence River (Quebec,
Canada). Ecoscience.

[PLS040C27] Jean M, Létourneau G (2011). Changements dans les milieux humides du fleuve Saint-Laurent de
1970–2002 [Changes in the wetlands of the St. Lawrence River,
1970–2002]. Science and Technology; Water Quality Monitoring—Quebec Region.

[PLS040C28] Jodoin Y, Lavoie C, Villeneuve P, Theriault M, Beaulieu J, Belzile F (2008). Highways as corridors and habitats for the invasive common reed
*Phragmites australis* in Quebec, Canada. Journal of Applied Ecology.

[PLS040C29] Keddy PA (2000). Wetland ecology principles and conservation.

[PLS040C30] Keller BEM (2000). Plant diversity in *Lythrum*, *Phragmites*,
and *Typha* marshes, Massachusetts, U.S.A. Wetlands Ecology and Management.

[PLS040C31] Kettenring KM, Mock KE (2012). Genetic diversity, reproductive mode, and dispersal differ between the
cryptic invader, *Phragmites australis*, and its native
conspecific. Biological Invasions.

[PLS040C32] Kettenring KM, Whigham DF (2009). Seed viability and seed dormancy of non-native *Phragmites
australis* in suburbanized and forested watersheds of the Chesapeake Bay,
USA. Aquatic Botany.

[PLS040C33] Kettenring KM, McCormick MK, Baron HM, Whigham DF (2010). *Phragmites australis* (common reed) invasion in the Rhode
River subestuary of the Chesapeake Bay: disentangling the effects of foliar nutrients,
genetic diversity, patch size, and seed viability. Estuaries and Coasts.

[PLS040C34] Kettenring KM, McCormick MK, Baron HM, Whigham DF (2011). Mechanisms of *Phragmites australis* invasion: feedbacks
among genetic diversity, nutrients, and sexual reproduction. Journal of Applied Ecology.

[PLS040C35] Kettenring KM, Garvie K, Hazelton ELG, Hough-Snee N, Ma Z (2012). *Phragmites* invasion and control in the Great Salt Lake
watershed: 2012 land manager survey.

[PLS040C36] King RS, DeLuca WV, Whigham DF, Marra PP (2007). Threshold effects of coastal urbanization on *Phragmites
australis* (common reed) abundance and foliar nitrogen in Chesapeake
Bay. Estuaries and Coasts.

[PLS040C37] Kirk H, Paul J, Straka J, Freeland JR (2011). Long-distance dispersal and high genetic diversity are implicated in the
invasive spread of the common reed, *Phragmites australis* (Poaceae), in
northeastern North America. American Journal of Botany.

[PLS040C38] Kulmatiski A, Beard KH, Meyerson LA, Gibson JR, Mock KE (2011). Nonnative *Phragmites australis* invasion into Utah
wetlands. Western North American Naturalist.

[PLS040C39] Lambertini C, Mendelssohn IA, Gustafsson MHG, Olesen B, Riis T, Sorrell BK, Brix H (2012). Tracing the origin of Gulf Coast *Phragmites* (Poaceae): a
story of long-distance dispersal and hybridization. American Journal of Botany.

[PLS040C40] Lavoie C, Jean M, Delisle F, Létourneau G (2003). Exotic plant species of the St Lawrence River wetlands: a spatial and
historical analysis. Journal of Biogeography.

[PLS040C41] **Le Groupe Phragmites** (2012). Le roseau envahisseur: la dynamique, l'impact et le contrôle
d'une invasion d'envergure. Naturaliste Canadien.

[PLS040C42] Lelong B, Lavoie C, Jodoin Y, Belzile F (2007). Expansion pathways of the exotic common reed (*Phragmites
australis*): a historical and genetic analysis. Diversity and Distributions.

[PLS040C43] Lodge DM, Williams S, Macisaac HJ, Hayes KR, Leung B, Reichard S, Mack RN, Moyle PB, Smith M, Andow DA, Carlton JT, McMichael A (2006). Biological invasions: recommendations for U.S. policy and
management. Ecological Applications.

[PLS040C44] Mack RN, Von Holle B, Meyerson LA (2007). Assessing invasive alien species across multiple spatial scales: working
globally and locally. Frontiers in Ecology and the Environment.

[PLS040C45] Maheu-Giroux M, de Blois S (2005). Mapping the invasive species *Phragmites australis* in
linear wetland corridors. Aquatic Botany.

[PLS040C46] Maheu-Giroux M, de Blois S (2007). Landscape ecology of *Phragmites australis* invasion in
networks of linear wetlands. Landscape Ecology.

[PLS040C47] Marks M, Lapin B, Randall J (1994). *Phragmites australis* (*Phragmites
communis*): threats, management, and monitoring. Natural Areas Journal.

[PLS040C48] McCormick MK, Kettenring KM, Baron HM, Whigham DF (2010). Extent and reproductive mechanisms of *Phragmites australis*
spread in brackish wetlands in Chesapeake Bay, Maryland (USA). Wetlands.

[PLS040C49] McCormick MK, Kettenring KM, Baron HM, Whigham DF (2010). Spread of invasive *Phragmites australis* in estuaries with
differing degrees of development: genetic patterns, Allee effects and
interpretation. Journal of Ecology.

[PLS040C50] Meadows RE, Saltonstall K (2007). Distribution of native and introduced *Phragmites australis*
in freshwater and oligohaline tidal marshes of the Delmarva peninsula and southern New
Jersey. Journal of the Torrey Botanical Society.

[PLS040C51] Metzgar RG (1973). Wetlands in Maryland.

[PLS040C52] Meyerson LA, Chambers RM, Vogt KA (1999). The effects of *Phragmites* removal on nutrient pools in a
freshwater tidal marsh ecosystem. Biological Invasions.

[PLS040C53] Meyerson LA, Lambert AM, Saltonstall K (2010). A tale of three lineages: expansion of common reed (*Phragmites
australis*) in the U.S. Southwest and Gulf Coast. Invasive Plant Science and Management.

[PLS040C54] Meyerson LA, Lambertini C, McCormick MK, Whigham DF (2012). Hybridization of common reed in North America? The answer is blowing in the
wind. AoB PLANTS **2012**: pls022; doi:10.1093/aobpla/pls022.

[PLS040C55] Minchinton TE, Bertness MD (2003). Disturbance-mediated competition and the spread of *Phragmites
australis* in a coastal marsh. Ecological Applications.

[PLS040C56] Minchinton TE, Simpson JC, Bertness MD (2006). Mechanisms of exclusion of native coastal marsh plants by an invasive
grass. Journal of Ecology.

[PLS040C57] Novak SJ, Mack RN (2001). Tracing plant introduction and spread: genetic evidence from *Bromus
tectorum* (cheatgrass). Bioscience.

[PLS040C58] Olson BE (2007). *Phragmites* control plan.

[PLS040C59] Olson BE, Lindsey K, Hirschboeck V (2004). Bear River Migratory Bird Refuge habitat management plan.

[PLS040C60] Pellegrin D, Hauber DP (1999). Isozyme variation among populations of the clonal species,
*Phragmites australis* (Cav.) Trin. ex Steudel. Aquatic Botany.

[PLS040C61] Rice D, Rooth JE, Stevenson JC (2000). Colonization and expansion of *Phragmites australis* in
upper Chesapeake Bay tidal marshes. Wetlands.

[PLS040C62] Saltonstall K (2002). Cryptic invasion by a non-native genotype of the common reed,
*Phragmites australis*, into North America. Proceedings of the National Academy of Sciences of the USA.

[PLS040C63] Saltonstall K, Peterson PM, Soreng RJ (2004). Recognition of *Phragmites australis* subsp.
*americanus* (Poaceae: Arundinoideae) in North America: evidence from
morphological and genetic analyses. SIDA.

[PLS040C64] Silliman BR, Bertness MD (2004). Shoreline development drives invasion of *Phragmites
australis* and the loss of plant diversity on New England salt
marshes. Conservation Biology.

[PLS040C65] Taddeo S, de Blois S (2012). Coexistence of introduced and native common reed (*Phragmites
australis*) in freshwater wetlands. Ecoscience.

[PLS040C66] Talley TS, Levin LA (2001). Modification of sediments and macrofauna by an invasive marsh
plant. Biological Invasions.

[PLS040C67] Tiner RW, Burke DG (1995). Wetlands of Maryland.

[PLS040C68] **Western Hemisphere Shorebird Reserve Network** (2009). www.whsrn.org.

[PLS040C69] Wijte AHBM, Gallagher JL (1996). Effect of oxygen availability and salinity on early life history stages of
salt marsh plants. I. Different germination strategies of *Spartina
alterniflora* and *Phragmites australis*
(Poaceae). American Journal of Botany.

[PLS040C70] Wijte AHBM, Gallagher JL (1996). Effect of oxygen availability and salinity on early life history stages of
salt marsh plants. II. Early seedling development advantage of *Spartina
alterniflora* over *Phragmites australis*
(Poaceae). American Journal of Botany.

[PLS040C71] Wilcox KL, Petrie SA, Maynard LA, Meyer SW (2003). Historical distribution and abundance of *Phragmites
australis* at Long Point, Lake Erie, Ontario. Journal of Great Lakes Research.

[PLS040C72] Windham L, Ehrenfeld JG (2003). Net impact of a plant invasion on nitrogen-cycling processes within a
brackish tidal marsh. Ecological Applications.

